# Reference Data and Predictors of HR‐pQCT‐Derived Muscle Density and Its Prediction of Physical Performance

**DOI:** 10.1002/jcsm.70029

**Published:** 2025-07-30

**Authors:** Stuart J. Warden, Ziyue Liu, Robyn K. Fuchs, Lilly G. Davisson, Keith G. Avin, Erik A. Imel, Kenneth Lim, Sharon M. Moe, Rachel K. Surowiec

**Affiliations:** ^1^ Department of Physical Therapy School of Health and Human Sciences, Indiana University Indianapolis Indianapolis Indiana USA; ^2^ Indiana Center for Musculoskeletal Health Indiana University School of Medicine Indianapolis Indiana USA; ^3^ Department of Biostatistics Indiana University School of Medicine Indianapolis Indiana USA; ^4^ Tom and Julie Wood College of Osteopathic Medicine Marian University Indianapolis Indiana USA; ^5^ Departments of Medicine and Pediatrics Indiana University School of Medicine Indianapolis Indiana USA; ^6^ Division of Nephrology and Hypertension, Department of Medicine Indiana University School of Medicine Indianapolis Indiana USA; ^7^ Weldon School of Biomedical Engineering Purdue University Indianapolis Indiana USA

**Keywords:** cachexia, computed tomography, muscle density, muscle quality, normative data, sarcopenia

## Abstract

**Background:**

There is increasing awareness of a role for muscle composition in sarcopenia and cachexia. Computed tomography (CT)–based measures of muscle density (MusD) are commonly used to indicate composition, with a decrease in MusD reflecting an increase in muscle fat infiltration. The current study explored predictors of MusD acquired using high‐resolution peripheral quantitative computed tomography (HR‐pQCT) and whether MusD predicted physical performance. In addition, reference data for MusD were generated and applied.

**Methods:**

HR‐pQCT scans performed in 1662 adults (aged 18–80 years) at 30% of bone length proximal from the distal end of the radius and tibia were analysed for forearm and leg MusD, respectively. Predictors of MusD were explored, and it was investigated whether MusD predicted physical performance. Centile curves were fit to the MusD data using the LMS approach to generate reference data, and a calculator was developed to enable computation of subject‐specific standardised outcomes. The utility of the calculator was explored in validation cohorts of female collegiate‐level athletes (*n* = 50) and individuals with chronic kidney disease (CKD) (*n* = 50).

**Results:**

Forearm and leg MusD were predicted by whole‐body percent fat, sex and age. Forearm and leg MusD were 0.46 (~1.9%) and 0.60 mgHA/cm^3^ (~2.6%) lower in females than in males, respectively (all *p* ≤ 0.002). For every decade of greater age, forearm and leg MusD were 0.28 (~1.2%) and 0.75 mgHA/cm^3^ (~3.3%) lower, respectively (all *p* < 0.001). These observations were independent of whole‐body percent fat and appendicular lean mass (ALM)/height^2^. MusD predicted grip strength, fast gait speed and self‐reported physical function independent of ALM/height^2^, body mass index and whole‐body percent fat. Grip strength was 0.756 kg (~2.4%) greater for every 1 mgHA/cm^3^ (~4.2%) greater forearm MusD (*p* < 0.001). Reference data were generated. Compared to the reference data, female athletes had above‐average leg MusD (*z*‐score = 0.20; 95% CI, 0.01–0.39), whereas those with CKD had *z*‐scores for forearm and leg MusD of −1.51 (95% CI, −1.95 to −1.08) and −1.70 (95% CI, −2.04 to −1.36), respectively.

**Conclusions:**

HR‐pQCT acquired MusD provides a novel indicator of muscle composition which predicts physical function independent of muscle quantity (i.e., ALM/height^2^). Whether the unique information provided by MusD has a role in quantifying health and the consequences of disease and illness (including sarcopenia and cachexia) requires further exploration. Studies in this area may be facilitated by the reference data generated which enable MusD in an individual or population of interest to be expressed relative to sex‐ and age‐matched norms.

## 1 Introduction

Sarcopenia and cachexia share phenotypes of reduced skeletal muscle strength and mass [1, 2]. Muscle mass predicts strength and is a determinant of morbidity and mortality [3]. However, muscle mass is an incomplete indicator of muscle health as it does not take into account the role of muscle composition, among other factors [4]. Aging and muscle wasting disorders are associated with changes in muscle composition, including the intra‐ and intermuscular accumulation of fat, which influences strength, morbidity and mortality independent of changes in muscle mass [5, 6, 7].

Muscle composition can be quantified using imaging modalities such as magnetic resonance imaging (MRI) and computed tomography (CT). MRI possesses the advantage of superior contrast resolution but presents challenges in terms of cost, accessibility and ease of image and data acquisition [8]. CT provides less granular information; however, it is popular due to its wider availability and because muscle can be assessed opportunistically on images acquired for other purposes [9, 10].

Muscle composition on CT is quantified by assessing x‐ray attenuation within a segmented muscle compartment, with attenuation data expressed in Hounsfield units (HUs) and typically scaled in relation to water (HU = 0) [9]. HU can subsequently be converted to density by scanning a phantom with known calcium hydroxyapatite concentrations. Changes in muscle composition are reflected by changes in CT‐derived muscle density (MusD). For example, fat infiltration into a muscle results in a decrease in MusD due to the lower density (i.e., x‐ray attenuation coefficient) of fat relative to muscle [9, 11, 12].

Opportunistic assessment of MusD via CT is often performed at the level of the third or fourth lumbar vertebrae as this location is frequently scanned during standard clinical care. However, abdominal CT scans are associated with a significant radiation dose (8 mSv for a typical abdomen/pelvis CT, equivalent to 3 years of background radiation) reducing their utility in screening otherwise healthy individuals. To minimise the radiation burden, low radiation dose peripheral quantitative computed tomography (pQCT) scanners are available to assess appendicular musculoskeletal properties. High‐resolution peripheral quantitative computed tomography (HR‐pQCT) is a relatively recent addition, imaging with an effective radiation dose of < 5 μSv and isotropic voxel size in the range of 60–80 μm [13].

The first‐generation HR‐pQCT scanner was developed to assess bone microarchitecture at peripheral sites rich in trabecular bone (e.g., distal radius and tibia). Soft tissue outcomes at these distal sites can be obtained [14], but the tissue is heterogeneous with a high proportion of tendinous structures. In contrast, the longer maximal scan length (22.5 vs. 15.0 cm) and larger field of view (14.0 vs. 12.6 cm) of the newer second‐generation scanner enable more muscle‐dominant proximal sites to be imaged. The site at 30% of bone length proximal from the distal end of the radius and tibia is recommended as it is accessible within the scan length (i.e., *z*‐axis) limits of the second‐generation scanner in most individuals when using the manufacturer's standard stabilisation casts and reference lines placed at the end of the bone [13]. An initial study reported MusD obtained by HR‐pQCT at the 30% tibia location independently predicted physical performance, mobility limitations, disability and mortality [15].

To enhance the utility of HR‐pQCT in assessing MusD, there is a need to explore contributing factors and generate reference data. We recently generated reference data for HR‐pQCT bone outcomes obtained at the 30% sites of the radius (i.e., forearm) and tibia (i.e., leg) [16, 17]. Using this repository of scans along with other scans we have performed, the aims of the current work were to (1) explore predictors of forearm and leg MusD, (2) generate reference data and develop a calculator to compute percentiles and *z*‐ and *t*‐scores for MusD, (3) apply the calculator to cohorts expected to have altered MusD (i.e., athletes and individuals with chronic kidney disease [CKD]) and (4) examine whether HR‐pQCT‐derived MusD independently predicts physical performance and function.

## 2 Methods

### 2.1 Participants

HR‐pQCT scans were performed on 2344 adults who underwent testing in our Function, Imaging, and Testing Resource Core (FIT Core) of the Indiana Center for Musculoskeletal Health's Clinical Research Center (Indianapolis, Indiana) between 12/2017 and 4/2024. Participants were recruited from the local community via self‐referral and by investigators seeking standardised musculoskeletal outcomes for their research participants. The FIT Core has Institutional Review Board approval from Indiana University to assess all‐comers (IU IRB Number: 1707550885). All individuals included in the current analyses provided written informed consent to participate and for their data to be utilised.

To be included in the cohort used to generate reference data and explore predictors, FIT Core participants were required to (1) be aged 18–80 years, (2) have at least one eligible forearm or leg HR‐pQCT scan, (3) be ambulatory and (4) have no self‐reported diabetes, liver or kidney disease, history of cancer, thyroid disorders, cystic fibrosis or rare bone disease (e.g., osteopetrosis or X‐linked hypophosphatemia). Individuals with the later conditions were excluded from the reference data cohort due to their overrepresentation in the FIT Core resulting from investigator‐initiated trials. Similarly, collegiate‐level athletes were omitted from the reference data cohort due to overrepresentation in the FIT Core. An age range of 18–80 years was selected because the FIT Core currently has limited participants beyond this range, with centile curves being sensitive to low numbers at the tail of distributions.

To explore the utility of the reference data generated, we used the data to calculate standardised outcomes (i.e., *z*‐scores) in validation cohorts consisting of individuals with CKD undergoing dialysis (Stage 5D) (*n* = 50) and female athletes competing in Division I, II or III cross‐country, track and field, tennis or soccer in the National Collegiate Athletic Association (*n* = 50). The latter groups of individuals were expected to have altered MusD and underwent testing in our FIT Core but were not included in the generation of the reference data (as detailed above).

### 2.2 Participant Characteristics

Height (metres) and weight (kilograms) were measured without shoes using a calibrated stadiometer (Seca 264; Seca GmbH & Co., Hamburg, Germany) and scale (MS140‐300; Brecknell, Fairmont, Minnesota), respectively. Whole‐body percent fat (percent) and appendicular lean mass relative to height squared (ALM/height^2^; kilograms per square metre) were measured by dual‐energy x‐ray absorptiometry (DXA) using either a Hologic Discovery‐W (Hologic Inc., Bedford, Massachusetts, United States), Hologic Horizon A (Hologic Inc., Bedford, Massachusetts, United States) or Norland Elite (Norland at Swissray, Fort Atkinson, WI) scanner. Data obtained using the Discovery‐W and Norland Elite scanners were converted to Hologic Horizon A equivalent values using regression formulae derived by scanning 30 individuals on each scanner.

Physical function was assessed using a standardised battery of tests. Maximum grip strength from three repeat efforts was assessed using a JamarPlus+ digital hand dynamometer (Sammons Preston, Bolingbrook, Illinois, United States) and the number of sit‐to‐stand manoeuvres completed in 30 s without the use of arms was assessed from a standardised seat (height = 45 cm), as we have previously described [18]. Pain limiting test performance was recorded using a 0–10 verbal rating scale.

Time to walk 4‐m from a stationary start at normal (usual gait speed) and fast (fast gait speed) speed was measured with a stopwatch and converted to speed (metre per second), as we have previously reported [19]. Self‐reported physical function was assessed using the physical function domain of the National Institutes of Health Patient‐Reported Outcomes Measurement Information System (PROMIS‐PF), performed via computerised adaptive testing. PROMIS scores are standardised and expressed as *t*‐scores with a population mean of 50 and standard deviation of 10 [20].

### 2.3 HR‐pQCT

A HR‐pQCT scanner (XtremeCT II; Scanco Medical AG, Bruttisellen, Switzerland) imaged the nondominant forearm and contralateral leg at 30% proximal from the distal end of the radius and tibia, respectively. The opposite arm or leg was imaged in the case of a previous fracture in the target limb. Phantoms were imaged daily to confirm scanner stability.

Radius and tibia length were measured using a segmometer (Realmet Flexible Segmometer, NutriActiva, Minneapolis, Minnesota). Ulna length (millimetres) was measured per convention as a surrogate for radial length. Scans were acquired and reconstructed as previously described [21]. Subjects lay supine with their limb immobilised using an anatomically formed carbon fibre cast. After performance of a scout view, reference lines were placed at the medial edge of the distal radius articular surface and centre of the distal tibia joint surface. Scans were performed at 68 kVp and 1.47 mA to acquire 168 slices (10.2 mm of bone length) with a voxel size of 60.7 μm. Scan stacks were centred 30% of bone length proximal to the reference lines. Scans were scored for motion artefacts on a standard scale of 1 (no motion) to 5 (discontinuities in the cortical shell) [22]. Scans scoring ≥ 3 were repeated when time permitted. Scans with a motion artefact of 4 or 5 were excluded from analyses.

The outer border of the limb (including skin) was contoured using a manufacturer‐developed script (Autocontour Limb; Scanco Medical AG). Soft tissue composition within the limb contour was subsequently assessed using a manufacturer‐provided evaluation script (Soft Tissue Analysis; Scanco Medical AG) with images filtered using a low‐pass Gaussian filter (sigma 0.8, support 1.0 voxel). The script was initially described by Erlandson et al. [14] for the first‐generation HR‐pQCT scanner and adapted to the second‐generation scanner by Hildebrand et al. [23].

**FIGURE 1:**
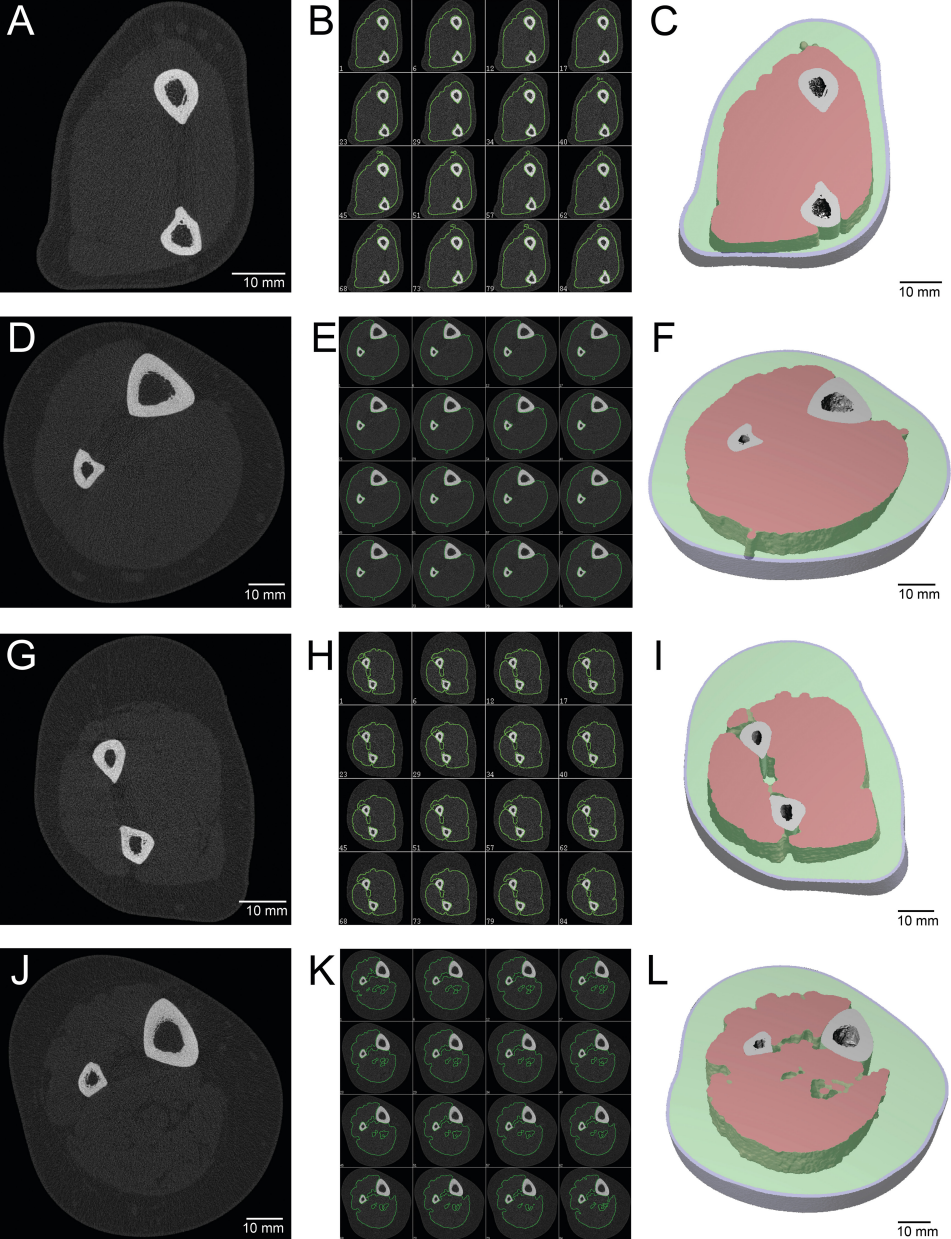
Representative HR‐pQCT images of the forearm (A–C) and leg (D–F) in a 44‐year‐old female from the reference cohort and the forearm (G–I) and leg (J–L) in a 52‐year‐old female with chronic kidney disease undergoing dialysis. The left panels (A, D, G, J) show a magnified single HR‐pQCT cross‐sectional slice. The middle panels (B, E, H, K) show every ~6^th^ slice of the downscaled images with the segmented muscle compartment contoured. The right panels (C, F, I, L) show three‐dimensional reconstructions of the analysed slices with the skin (outer 20 voxels; blue) and subcutaneous fat (green) shown in transparent and the muscle compartment shown in red. The 44‐year‐old reference cohort participant (A–F) had forearm and leg muscle density of 24.10 (*z*‐score = 0.4) and 22.36 mgHA/cm^3^ (*z*‐score = −0.3), respectively. The 52‐year‐old with chronic kidney disease (G–L) had forearm and leg muscle density of 20.96 (*z*‐score = −1.9) and 16.71 mgHA/cm^3^ (*z*‐score = −2.5), respectively.

The soft tissue analysis script downscales the images by a factor of two (from 60.7 to 121.4 μm resolution) to reduce noise and image processing time. The skin layer (outer 20 voxels) and bone tissue (including arterial calcifications) are segmented via thresholding and excluded from analysis. Muscle and adipose tissue (both subcutaneous and intermuscular fat) are subsequently identified using an iterative method. Seed volumes are planted in muscle and adipose tissue by applying thresholds of 100–600 HU and −600 to −200 HU, respectively. Seed volumes less than 50 voxels are removed, as these unconnected small islands of voxels are likely noise. A total of 10 iterations are performed, with seed volumes dilated by one voxel in each iteration. Example leg HR‐pQCT images and muscle compartment segmentations are shown in Figure 1.

MusD was quantified by converting linear attenuation coefficients to mgHA/mm^3^ using linear grayscale‐to‐density calibration coefficients derived from a standardised phantom of known density. Total volume (TV; cubic centimetre), muscle volume (MV; cubic centimetre) and muscle volume fraction (MV/TV; percent) were also recorded. Short‐term precision on triplicate forearm and leg scans with interim repositioning in 10 individuals showed root mean square coefficients of variation of ≤ 0.6% and ≤ 1.5% for MV/TV and MusD, respectively.

### 2.4 Statistical Analyses

Participant characteristics in the reference cohort were described according to decade of life (18–29, 30–39, 40–49, 50–59, 60–69 and 70–80 years). Age and sex were not evenly distributed across races (Asian vs. Black or African American vs. White vs. other) and ethnicities (Hispanic or Latino vs. non‐Hispanic or Latino), so predictors of MusD were explored using multivariable linear regression models, and adjusted *p*‐values were reported. Potential predictors explored, based on biological plausibility, were age, sex, race, ethnicity, height, weight, BMI, whole body percent fat and ALM/height^2^. Variation inflation factors (VIFs) were used to assess collinearity between predictors.

Sex‐specific reference centile curves for MusD were generated using the LMS method [24] with R package GAMLSS (Version 5.2.0) [25]. The LMS method uses Box–Cox transformation to achieve normality at a given age (Box–Cox Cole and Green [BCCG] distribution). Nonparametric smooth curves are fit to the parameter values across the age range using penalised likelihood with penalty on the second derivatives.

Centile curves, percentiles and *z*‐ and *t*‐scores were calculated from the estimated parameter curves. *z*‐scores represent the number of standard deviations the subject‐specific MusD varies from age‐ and sex‐matched median outcomes. *t*‐scores represent the number of standard deviations the subject‐specific MusD varies from the best sex‐specific median outcome. The best sex‐specific MusD median outcome was determined as the highest point on the fitted median curve, where the age and corresponding standard deviation were identified.

LMS‐derived *z*‐scores and *t*‐scores are not suited for identifying extreme values because the LMS transformation method to achieve normality constrains maximum obtainable scores. Modified *z*‐scores and *t*‐scores are provided for scores greater than +2 to address this. In modified *z*‐scores, the HR‐pQCT outcome is expressed relative to the sex‐ and age‐matched median in units of half the distance between 0 and +2 *z*‐scores, as per the approach used for growth charts [26]. Modified *t*‐scores are expressed in units of half the distance between 0 and +2 *z*‐scores at the age of best sex‐specific median outcome in the database.

The data from the reference cohort were used to compute *z*‐ and *t*‐scores for MusD acquired from individuals with CKD (Stage 5D) and female collegiate‐level athletes. Mean and 95% confidence intervals (95% CI) not crossing zero indicated differences from the reference cohort, as determined by single sample *t*‐tests with a population mean of 0 and a level of significance set at 0.05.

MusD in the reference cohort was explored as an independent predictor of physical function (usual and fast gait speed, grip strength, 30‐s sit‐to‐stand test performance and PROMIS‐PF score) by adding each MusD to base multivariable linear regression models containing age, sex, race, ethnicity, height, BMI, whole‐body percent fat and ALM/height^2^.

## 3 Results

### 3.1 Reference Cohort Participant and Scan Characteristics

Scans from 1662 participants were included in the reference data cohort following exclusion of 672 participants due to (1) age > 80 years (*n* = 49), (2) a self‐reported ineligible disease or illness (*n* = 323), (3) being nonambulatory (*n* = 19) and (4) absence of at least one eligible HR‐pQCT scan at the 30% forearm or leg site (*n* = 281). The final reference cohort included 1161 females and 501 males ranging in age from 18.5 to 79.9 years and 18.4 to 80.0 years, respectively.

Participant characteristics stratified by decade of age are detailed in Table 1. MusD in females (age range: 18.5–79.9 years) was acquired from 1143 and 1000 scans of the forearm and leg, respectively. MusD in males (age range: 18.2–80.0 years) was acquired from 476 and 407 scans of the forearm and leg, respectively. Lower scan numbers than participants were due to a combination of (1) scan not performed due to time constraints, (2) excessive motion artefact and (3) participant size (e.g., leg too large resulting in the generation of a local tomography artefact due to absorbing tissue out of the scanner's 14.0 cm field of view; leg too long to place both the reference line and scan stack within the constraints of the scanner's 22.5 cm long *z*‐axis).

**TABLE 1:** Participant characteristics stratified by decade of age^a^.

Characteristic	Age group (years)					
	18–29	30–39	40–49	50–59	60–69	70–80
**Females**						
*n*	206	125	129	263	322	116
Race (*n*)						
Asian	25 (12.1%)	16 (12.8%)	9 (7.0%)	11 (4.2%)	6 (1.9%)	3 (2.6%)
Black or African American	18 (8.7%)	11 (8.8%)	8 (6.2%)	25 (9.5%)	26 (8.1%)	10 (8.6%)
White	160 (77.7%)	97 (77.6%)	110 (85.3)	226 (85.9)	290 (90.1%)	102 (87.9%)
Other	3 (1.5%)	1 (0.8%)	2 (1.6%)	1 (0.4%)	0 (0%)	1 (0.9%)
Ethnicity (*n*)						
Non‐Hispanic or Latino	193 (93.7%)	119 (95.2%)	126 (97.7%)	256 (97.3%)	317 (98.4%)	116 (100%)
Hispanic or Latino	13 (6.3%)	6 (4.8%)	3 (2.3%)	7 (2.7%)	5 (1.6%)	0 (0%)
Height (m)	1.64 (1.60–1.69)	1.65 (1.60–1.70)	1.65 (1.61–1.68)	1.63 (1.59–1.67)	1.63 (1.59–1.67)	1.61 (1.58–1.65)
Weight (kg)	66.5 (58.9–76.4)	67.6 (60.6–82.0)	69.7 (60.5–86.1)	70.2 (59.4–87.2)	71.0 (62.1–84.2)	66.9 (59.6–81.2)
BMI (kg/m^2^)	24.6 (21.8–28.0)	24.6 (22.0–30.5)	26.0 (22.6–31.9)	26.0 (22.9–32.4)	26.3 (23.2–31.4)	26.0 (22.4–30.3)
Whole‐body percent fat (%)	30.7 (25.2–35.1)	31.2 (26.0–36.9)	33.4 (26.9–37.8)	36.0 (30.8–40.4)	35.9 (32.1–39.6)	35.7 (31.6–40.3)
ALM/height^2^ (kg/m^2^)	7.05 (6.21–7.91)	6.88 (6.22–7.93)	6.86 (5.99–7.67)	6.55 (5.83–7.71)	6.39 (5.84–7.34)	6.08 (5.61–6.83)
Physical function						
Usual gait speed (m/s)	1.41 (1.26–1.55)	1.39 (1.26–1.55)	1.44 (1.32–1.62)	1.43 (1.27–1.59)	1.35 (1.20–1.52)	1.30 (1.16–1.43)
Fast gait speed (m/s)	2.04 (1.84–2.21)	1.96 (1.79–2.19)	2.03 (1.83–2.17)	1.91 (1.75–2.11)	1.85 (1.70–2.00)	1.74 (1.55–1.94)
Grip strength (kg)	27.0 (23.5–31.2)	28.3 (25.0–32.1)	27.2 (22.8–31.3)	24.7 (21.2–28.2)	23.9 (20.8–27.0)	22.0 (17.9–25.4)
30‐s sit‐to‐stand test (n)	16 (14–19)	16 (13–19)	16 (13–18)	15 (13–18)	14 (12–16)	13 (11–16)
PROMIS‐PF (T‐score)	57.9 (52.4–62.8)	55.8 (51.3–61.9)	53.6 (48.7–60.1)	51.7 (46.8–57.3)	50.4 (45.9–54.7)	48.8 (44.7–53.9)
HR‐pQCT scans included (*n*)						
Forearm	204	124	127	259	316	113
Leg	182	112	118	215	272	101
**Males**						
*n*	145	61	48	64	110	73
Race (*n*)						
Asian	21 (14.5%)	6 (9.8%)	4 (8.3%)	3 (4.7%)	3 (2.7%)	2 (2.7%)
Black or African American	8 (5.5%)	4 (6.6%)	3 (6.3%)	10 (15.6%)	5 (4.5%)	3 (4.1%)
White	115 (79.3%)	51 (83.6%)	41 (85.4%)	51 (79.7%)	102 (92.7%)	68 (93.2%)
Other	1 (0.7%)	0 (0%)	0 (0%)	0 (0%)	0 (0%)	0 (0%)
Ethnicity (*n*)						
Non‐Hispanic or Latino	132 (91.0%)	53 (86.9%)	43 (89.6%)	63 (98.4%)	106 (96.4%)	73 (100%)
Hispanic or Latino	13 (9.0%)	8 (13.1%)	5 (10.4%)	1 (1.6%)	4 (3.6%)	0 (0%)
Height (m)	1.78 (1.74–1.78)	1.77 (1.73–1.81)	1.78 (1.73–1.84)	1.76 (1.71–1.84)	1.77 (1.73–1.81)	1.75 (1.70–1.79)
Weight (kg)	81.6 (72.2–91.5)	81.1 (74.3–95.5)	90.1 (78.9–102.2)	86.4 (75.5–100.4)	84.7 (76.6–96.3)	87.4 (76.8–99.6)
BMI (kg/m^2^)	25.3 (23.5–28.6)	25.7 (24.5–30.8)	27.6 (24.5–31.5)	27.2 (24.7–32.3)	27.6 (24.3–30.3)	28.3 (25.2–32.6)
Whole‐body percent fat (%)	18.6 (14.8–25.1)	20.9 (17.3–24.7)	22.9 (17.4–26.5)	23.8 (19.9–28.3)	24.7 (20.7–28.8)	28.2 (24.1–31.3)
ALM/height^2^ (kg/m^2^)	8.98 (8.11–9.72)	8.71 (8.03–9.78)	8.92 (8.19–10.48)	8.71 (8.12–10.27)	8.06 (7.57–8.82)	7.93 (7.18–8.80)
Physical function						
Usual gait speed (m/s)	1.44 (1.33–1.60)	1.42 (1.27–1.65)	1.44 (1.28–1.58)	1.42 (1.27–1.52)	1.39 (1.27–1.54)	1.29 (1.13–1.46)
Fast gait speed (m/s)	2.14 (1.97–2.35)	2.26 (1.99–2.41)	2.14 (1.81–2.39)	2.04 (1.86–2.33)	2.03 (1.85–2.24)	1.87 (1.61–2.12)
Grip strength (kg)	48.0 (40.2–55.3)	46.7 (39.2–56.9)	50.3 (41.2–55.4)	45.1 (37.6–48.6)	40.8 (31.9–48.2)	31.8 (27.0–40.5)
30‐s sit‐to‐stand test (n)	17 (15–21)	18 (15–22)	17 (14–20)	16 (14–19)	10.3 (8.6–11.5)	13 (11–16)
PROMIS‐PF (*T*‐score)	60.4 (55.8–64.7)	60.1 (53.6–66.6)	54.3 (49.8–62.7)	53.5 (48.6–60.5)	50.5 (47.1–54.7)	48.6 (45.7–51.7)
HR‐pQCT scans included (*n*)						
Forearm	139	59	44	61	104	69
Leg	121	51	37	53	88	57

Abbreviations: ALM/height^2^, appendicular lean mass relative to height squared; BMI, body mass index; PROMIS‐PF, physical function domain of the National Institutes of Health Patient‐Reported Outcomes Measurement Information System.

^a^ Data are median (interquartile range), except for frequencies.

### 3.2 Predictors of MusD

MusD (and MV, TV and MV/TV) in the reference cohort stratified by decade of age are detailed in Table 2. Height, weight and BMI were collinear, with VIFs of 25.6, 149.2 and 95.0, respectively. Removal of weight as a predictor variable reduced VIFs for remaining variables to < 10.

**TABLE 2:** HR‐pQCT muscle characteristics stratified by decade of age^a^.

Characteristic	Age group (years)					
	18–29	30–39	40–49	50–59	60–69	70–80
**Females**						
*Forearm*						
MV (cm^3^)	14.2 (12.7, 16.3)	14.8 (13.2, 17.1)	14.8 (13.1, 16.5)	14.2 (12.8, 16.1)	13.9 (12.6, 15.3)	13.3 (12.1, 14.7)
TV (cm^3^)	19.9 (17.4, 22.8)	20.0 (17.7, 22.7)	20.1 (18.0, 23.0)	19.7 (17.7, 23.0)	19.4 (17.4, 21.7)	18.9 (16.9, 21.0)
MV/TV (%)	72.6 (68.7, 76.2)	74.5 (69.6, 77.3)	72.8 (68.2, 77.0)	71.3 (67.9, 75.5)	71.4 (67.3, 75.8)	72.4 (67.4, 75.8)
MusD (mgHA/cm^3^)	23.91 (23.72, 24.10)	23.65 (23.42, 23.87)	23.74 (23.50, 23.98)	23.13 (22.94, 23.33)	22.65 (22.48, 22.82)	22.10 (21.82, 22.38)
*Leg*						
MV (cm^3^)	27.1 (23.2, 30.9)	26.8 (23.1, 29.9)	28.0 (24.2, 32.9)	26.7 (23.6, 30.6)	26.4 (23.7, 29.9)	24.6 (22.8, 27.3)
TV (cm^3^)	39.7 (34.6, 46.3)	39.3 (34.5, 45.4)	39.7 (35.8, 47.5)	40.3 (35.4, 45.6)	38.9 (35.0, 44.5)	37.0 (32.9, 41.6)
MV/TV (%)	67.0 (64.5, 70.2)	68.2 (65.1, 70.9)	68.4 (63.7, 71.5)	67.7 (62.9, 71.3)	67.8 (63.8, 71.6)	67.7 (63.1, 72.8)
MusD (mgHA/cm^3^)	24.08 (23.85, 24.30)	23.56 (23.22, 23.89)	22.86 (22.54, 23.17)	21.56 (21.30, 21.82)	20.51 (20.27, 20.75)	20.07 (19.65, 20.49)
**Males**						
*Forearm*						
MV (cm^3^)	22.3 (19.8, 25.2)	21.7 (19.0, 25.4)	24.5 (20.6, 28.2)	22.7 (20.5, 26.6)	20.8 (19.0, 23.0)	20.1 (17.4, 22.7)
TV (cm^3^)	27.7 (24.7, 30.9)	27.3 (23.6, 32.5)	30.2 (26.1, 35.2)	28.7 (26.1, 33.5)	26.8 (23.8, 30.4)	26.5 (22.5, 30.3)
MV/TV (%)	81.9 (76.9, 86.7)	80.2 (76.1, 84.7)	80.0 (74.5, 85.4)	80.4 (76.0, 83.4)	78.0 (74.6, 82.9)	76.4 (72.9, 81.2)
MusD (mgHA/cm^3^)	24.75 (24.53, 24.97)	24.70 (24.42, 24.97)	24.55 (24.09, 25.01)	24.39 (23.99, 24.79)	24.28 (23.99, 24.57)	23.48 (23.05, 23.91)
*Leg*						
MV (cm^3^)	31.9 (28.4, 36.5)	33.2 (30.0, 38.0)	33.3 (29.0, 36.0)	33.2 (31.6, 38.3)	31.9 (28.7, 35.4)	30.3 (27.1, 35.0)
TV (cm^3^)	43.0 (39.3, 49.5)	44.0 (37.1, 50.5)	42.7 (38.9, 47.0)	42.4 (40.3, 50.7)	42.7 (37.6, 46.9)	43.5 (36.5, 47.9)
MV/TV (%)	75.0 (70.1, 78.6)	76.3 (72.5, 80.0)	77.3 (71.8, 81.5)	78.5 (74.0, 81.8)	76.0 (71.7, 79.5)	75.6 (67.0, 78.9)
MusD (mgHA/cm^3^)	25.64 (25.39, 25.89)	24.61 (24.15, 25.07)	24.19 (23.53, 24.85)	23.54 (22.91, 24.17)	22.29 (21.86, 22.72)	21.50 (20.88, 22.12)

Abbreviations: MusD, muscle density; MV, muscle volume; MV/TV, muscle volume/total volume; TV, total volume.

^a^ Data are median (interquartile range).

Predictors of forearm and leg MusD are shown in Table 3. Predictors of forearm and leg MV, TV and MV/TV are available in File S1. Being female and having a greater age and whole‐body percent body fat were independently associated with a lower MusD at both the forearm and leg (all *p* ≤ 0.02). ALM/height^2^ and race and ethnicity were not associated with MusD (all *p* = 0.07–0.85).

**TABLE 3:** Predictors of forearm and leg MusD (mgHA/cm^3^)^a^.

Predictor	Forearm MusD				Leg MusD			
	Estimate	SE	Adj. *p*	*R* ^2^	Estimate	SE	Adj. *p*	*R* ^2^
Age (year)	−0.028	0.002	< 0.001*	0.275	−0.075	0.003	< 0.001*	0.485
Sex (female vs. male)	−0.458	0.131	< 0.001*		−0.595	0.195	0.002*	
Race								
Asian vs. White	−0.242	0.143	0.09		−0.181	0.212	0.39	
Black vs. White	0.047	0.134	0.73		−0.227	0.205	0.27	
Others vs. White	0.088	0.468	0.85		0.780	0.645	0.23	
Ethnicity (Hispanic vs. non‐Hisp.)	−0.238	0.173	0.17		−0.206	0.254	0.42	
Height (cm)	0.024	0.005	< 0.001*		0.012	0.008	0.14	
BMI (kg/m^2^)	0.038	0.017	0.02*		−0.046	0.027	0.09	
Whole‐body percent fat (%)	−0.022	0.010	0.02*		−0.051	0.015	< 0.001*	
ALM/height^2^ (kg/m^2^)	0.052	0.056	0.36		0.161	0.088	0.07	

Abbreviations: ALM/height^2^, appendicular lean mass relative to height squared; BMI, body mass index; MusD, muscle density; SE, stardard error.

^a^ Determined using multivariable linear regression.

* *p* < 0.05.

### 3.3 Centile Curves

**FIGURE 2:**
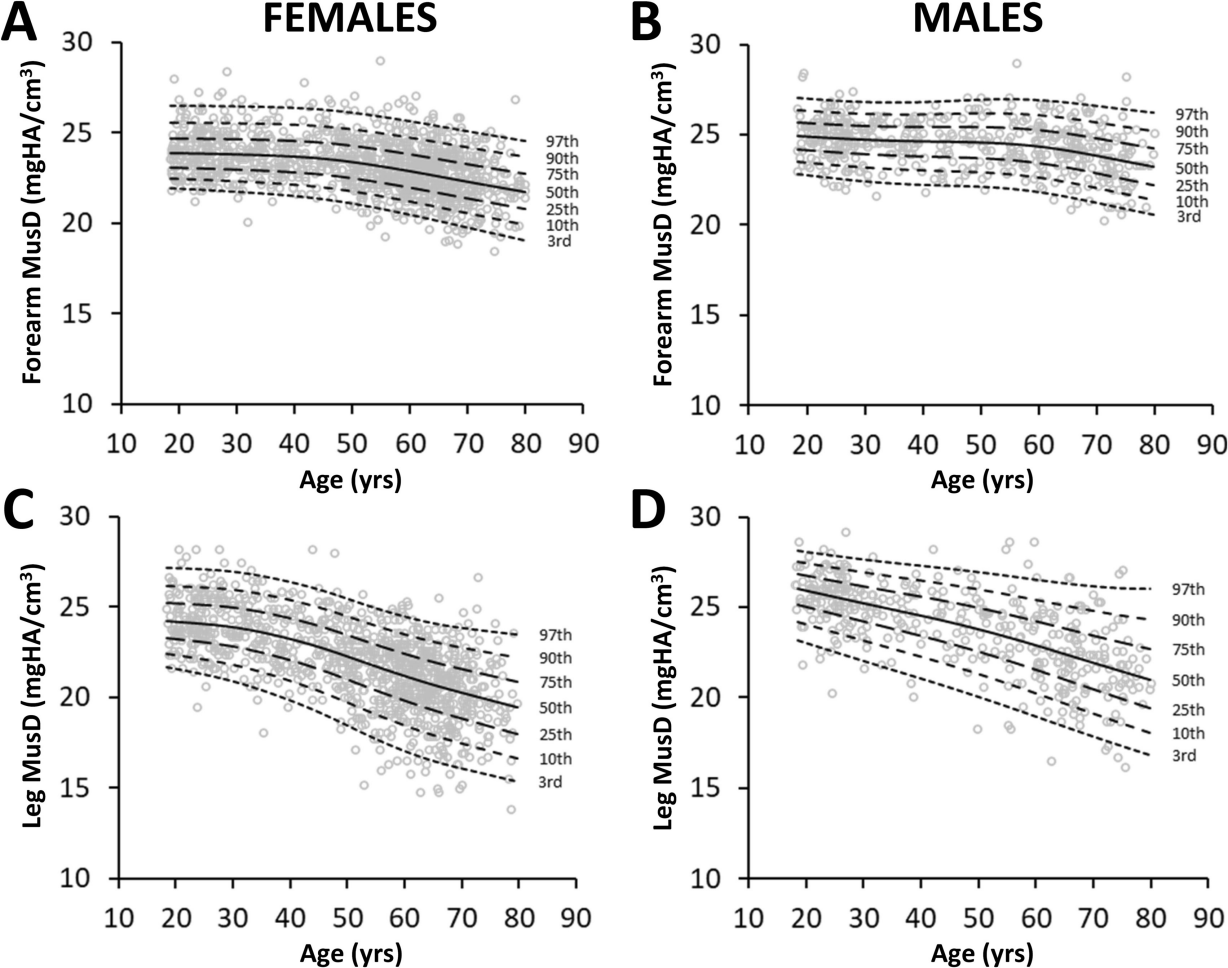
Raw data and fitted centile curves for forearm (A, B) and leg (C, D) muscle density (MusD) in females (A, C) and males (B, D).

Sex‐specific centile curves were generated as sex strongly predicted MusD. The fitted median centile curves for both forearm and leg MusD in both females and males were highest at the youngest age in the cohort and declined thereafter (Figure 2). For forearm MusD, the fitted median centile curve in females and males was 0.34 (1.4%) and 0.28 mgHA/cm^3^ (1.1%) lower per decade from peaks of 23.85 and 24.90 mgHA/cm^3^, respectively. For leg MusD, the fitted median centile curve in females and males was 0.78 (3.2%) and 0.82 mgHA/cm^3^ (3.2%) lower per decade from peaks of 24.20 and 26.06 mgHA/cm^3^, respectively. Fitted centile curves for MV, TV and MV/TV are available in File S2.

### 3.4 Calculator for Percentiles, *z*‐Scores, *t‐*Scores and Centile Curve Plotter

**FIGURE 3:**
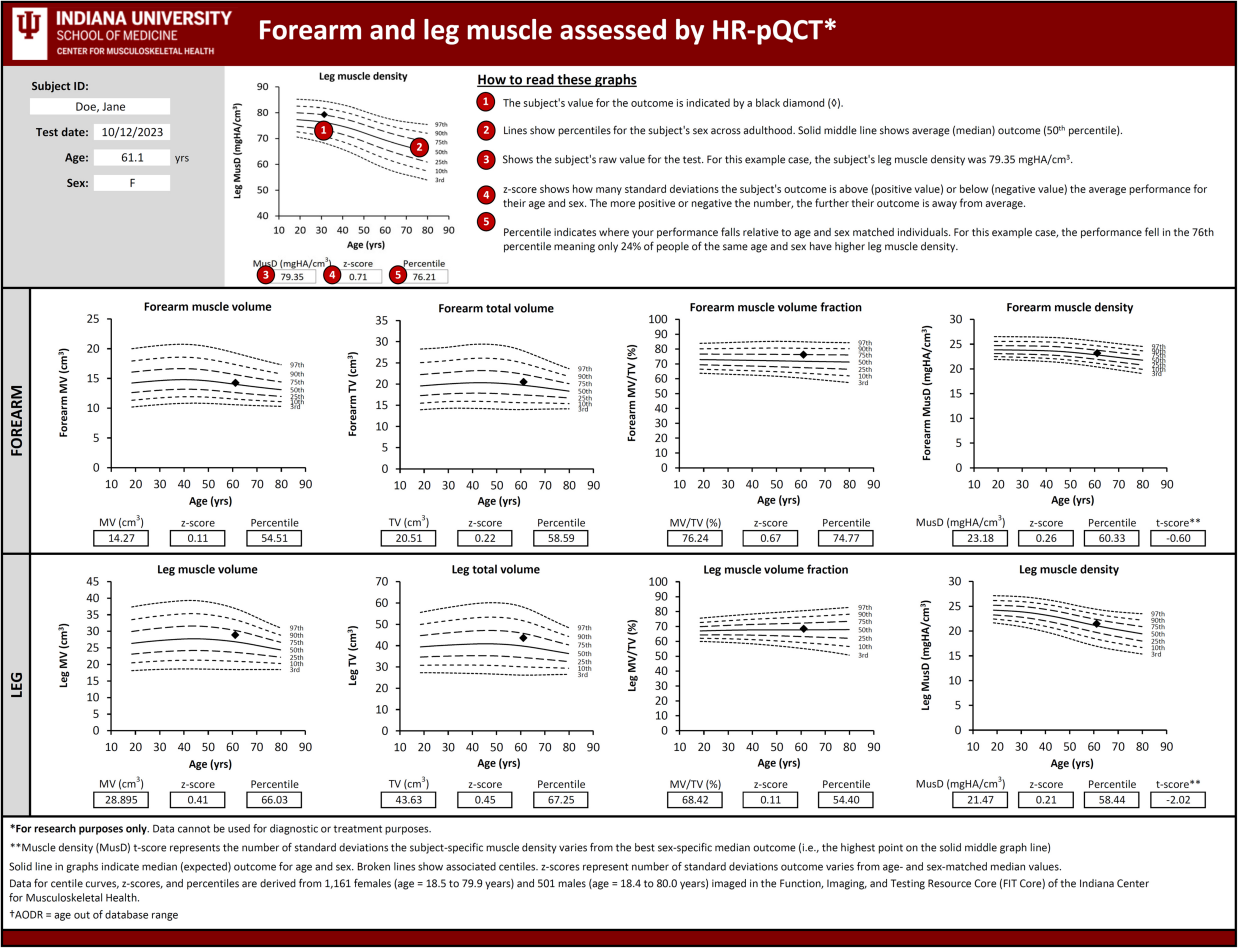
Screenshot of the Excel‐based calculator for calculating subject‐specific percentiles, *z*‐scores and *t*‐scores. The calculator is available as File S3.

An Excel‐based calculator for comparing an individual's MusD (and MV, TV and MV/TV) to the reference cohort was developed and is available in File S3. Entry of an individual's basic demographic information (sex, date of birth and scan date) and one or more HR‐pQCT outcomes results in the calculation of subject‐specific percentiles, *z*‐scores and *t*‐scores (Figure 3). The percentiles, *z*‐scores and *t*‐scores are derived from the centile curves fitted to the reference data using the LMS approach. The calculator enables data to be entered and percentiles, *z*‐scores and *t*‐scores to be calculated for up to 10 individuals at a time. In addition, the calculator plots sex‐specific centile curves for the individual whose data are entered in the first row on the data entry worksheet.

### 3.5 Utility of the Reference Data and Calculator

Utility of the reference data and calculator were explored using HR‐pQCT scans of the forearm and leg from female collegiate‐level athletes (*n* = 50 [*n* = 12 cross‐country, *n* = 11 softball, *n* = 15 soccer, *n* = 12 tennis athletes]; age = 20.4 ± 1.1 years; height = 1.68 ± 0.08 m; weight = 67.1 ± 11.9 kg; BMI = 23.6 ± 3.1 kg/m^2^; whole‐body percent fat = 23.6 ± 5.4%; ALM/height^2^=7.5 ± 0.8 kg/m^2^) and individuals with CKD undergoing dialysis (*n* = 50 [19 females, 31 males]; age = 52.2 ± 12.8 years; height = 1.69 ± 0.10 m; weight = 83.1 ± 15.9 kg; BMI = 29.1 ± 6.1 kg/m^2^; whole‐body percent fat = 30.9 ± 9.5%; ALM/height^2^ = 8.1 ± 1.4 kg/m^2^).

**FIGURE 4:**
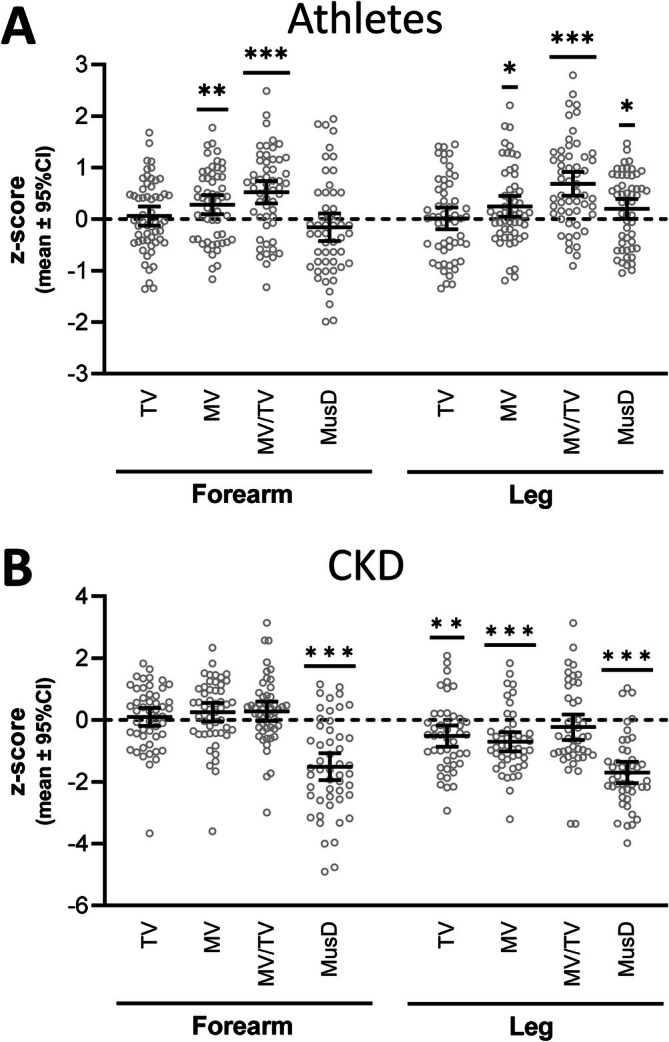
*z*‐scores (mean ± 95% confidence interval) for muscle density (MusD) (and total volume [TV], muscle volume [MV] and muscle volume fraction [MV/TV]) in female collegiate‐level athletes (A) and individuals with Stage 5 chronic kidney disease (CKD) (B). *p < 0.05, **p < 0.01 and ***p < 0.001, as determined by simple sample *t*‐test with a population mean of 0.

MusD (and MV, TV and MV/TV) data were entered into the calculator to derive standardised scores. The 95% CI for MusD *z*‐scores at the forearm in athletes crossed zero (*p* = 0.25); however, leg MusD in athletes was 0.20 standard deviations (95% CI, 0.01–0.39) greater than in the reference cohort (Figure 4A). The age of the athletes was around the age of peak median MusD, so their *t*‐scores do not vary greatly from *z*‐scores and are not reported. Individuals with CKD had *z*‐scores for forearm and leg MusD that were −1.51 (95% CI, −1.95 to −1.08) and −1.70 (95% CI, −2.04 to −1.36), respectively (Figure 4B). Forearm and leg MusD *t*‐scores in individuals with CKD were −2.48 (95% CI, −3.07 to −1.89) and −3.83 (95% CI, −4.44 to −3.22), respectively.

### 3.6 MusD Independently Predicts Physical Performance and Function

In the reference cohort, grip strength and 30‐s sit‐to‐stand tests limited by pain (> 3‐out‐of‐10, verbal reporting scale) were omitted from analyses. This included 19 grip strength tests in 16 females and three males and 61 sit‐to‐stand tests in 45 females and 16 males.

Base model variables (age, sex, race, ethnicity, height, BMI, whole‐body percent body fat and ALM/height^2^) explained approximately one‐quarter of the variance in fast gait speed (22.8%) and sit‐to‐stand manoeuvers completed in 30 s (25.9%), over one‐third (37.4%) of variance in self‐reported physical function (PROMIS‐PF) and nearly two‐thirds (64.8%) of variance in grip strength (Table 4).

**TABLE 4:** Prediction of physical performance and function by MusD (mgHA/cm^3^)^a^.

Physical function outcome	Base model^b^	Base model + forearm MusD				Base model + leg MusD			
	*R* ^2^	*R* ^2^	Estimate^c^	SE^c^	Adj. *p* ^c^	*R* ^2^	Estimate^c^	SE^c^	Adj. *p* ^c^
Usual gait speed (m/s)	0.128	0.128	−0.001	0.005	0.98	0.129	0.005	0.003	0.10
Fast gait speed (m/s)	0.228	0.230	0.009	0.006	0.12	0.231	0.009	0.004	0.03*
Grip strength (kg)	0.648	0.656	0.756	0.143	< 0.001*	0.649	0.211	0.105	0.04*
30‐s sit‐to‐stand test (*n*)	0.259	0.261	0.134	0.077	0.08	0.261	0.096	0.056	0.09
PROMIS‐PF (*T*‐score)	0.374	0.375	0.200	0.138	0.15	0.376	0.212	0.100	0.03*

Abbreviations: MusD, muscle density; PROMIS‐PF, physical function domain of the National Institutes of Health Patient Reported Outcomes Measurement Information System; SE, standard error.

^a^ Determined using multivariable linear regression;

^b^ Base model predictors included were age, sex, race, ethnicity, height, BMI, whole‐body percent fat, and appendicular lean mass/height^2^

^c^ Estimate, SE and adjusted (adj.) *p* value are for HR‐pQCT soft tissue composition after controlling for the influence of base model predictors.

* *p* < 0.05.

Forearm and leg MusD both positively predicted grip strength over and above the variables included in the base model (Table 4). Grip strength was 0.756 (~2.4%) and 0.211 kg (~0.7%) greater for every 1 mgHA/cm^3^ greater MusD at the forearm (~4.2%) and leg (~4.4%), respectively (all *p* ≤ 0.04). Leg MusD also positively predicted fast gait speed and self‐reported physical function (PROMIS‐PF). For every 1 mgHA/cm^3^ greater leg MusD (~4.4%), fast gait speed and self‐reported physical function were 0.009 m/s (~0.5%) and 0.212 (~0.4%) greater, respectively (all *p* = 0.03). Similar findings were observed for forearm and leg MV/TV, albeit with lower estimate values (File S4). MV/TV at both sites predicted grip strength over and above the variables included in the base model, with leg MV/TV also predicting usual and fast gait speed.

## Discussion

The current study provides novel observations and reference data for MusD acquired using a second‐generation HR‐pQCT scanner, expanding on prior studies reporting soft tissue outcomes with HR‐pQCT [14, 15, 23, 27]. MusD at both the forearm and leg sites was predicted by whole‐body percent fat, sex and age, and MusD predicted physical performance. These observations were independent of ALM/height^2^, indicating that HR‐pQCT‐derived MusD provides additional information beyond this conventional indicator of muscle quantity. Reference data generated from centile curves fitted to data from over 1100 females and 500 males were used to develop a calculator that can compute subject‐specific percentiles and *z*‐ and *t*‐scores. To demonstrate the utility of the calculator, we showed that collegiate‐level athletes and individuals with CKD (Stage 5) had elevated and reduced *z*‐scores for MusD, respectively.

The current data support MusD as an indicator of muscle composition that is independent of muscle quantity. Whole‐body percent fat predicted MusD independent of ALM/height^2^ and BMI, with MusD being 0.11 (~0.5%) and 0.26 mgHA/cm^3^ (~1.1%) lower in the forearm and leg for every five‐unit (5%) greater whole‐body percent fat, respectively. The lower MusD with greater whole‐body percent fat is consistent with work demonstrating that general adiposity results in fat infiltration of skeletal muscle [28, 29] and supports CT‐based MusD as one of the most common assessments of muscle fat infiltration [30]. We did not assess whether the fat was located inter‐ versus intramuscular, although this may be possible using more advanced segmentation and analysis approaches [9, 27]. We also did not assess the inter‐ vs. intramyocellular distribution of the fat as this requires the use of alternate techniques, such as magnetic resonance spectroscopy or tissue biopsy.

Sex predicted MusD, with females having 0.46 (~1.9%) and 0.60 mgHA/cm^3^ (~2.6%) lower forearm and leg MusD than males, respectively. Females have been shown to have lower MusD than males when assessed at other locations and using alternate CT imaging techniques [31, 32]. Females may be predicted to have lower MusD than males due to generally higher whole‐body percent fat. Accordingly, Straight et al. [33] reported that higher general body adiposity accounted for the greater lipid content they observed in the quadriceps of older females. However, sex differences in the current study were independent of whole‐body percent fat, BMI and ALM/height^2^, suggesting that factors other than general adiposity and muscle quantity contribute to sex dimorphism in skeletal muscle composition [34].

For every decade of greater age, MusD was 0.28 (1.2%) and 0.75 mgHA/cm^3^ (3.3%) lower in the forearm and leg, respectively. The age‐related lower MusD was independent of overall body composition (i.e., ALM/height^2^, BMI and whole‐body percent fat), indicating that aging itself is associated with muscle compositional changes. Possible underlying contributors include age‐related motoneuron and neuromuscular changes, changes in muscle microvasculature and mitochondrial function and muscle underuse, which combine to lower muscle protein synthesis and lead to the replacement of higher density contractile material with lower density fat [35, 36]. The 1%–3% less MusD we observed per decade of aging is lower than the 4%–34% observed by Johannesdottir et al. [31]; however, the use of different modalities (HR‐pQCT vs. conventional abdominal CT) and assessment locations (forearm/leg vs. trunk muscles) makes between‐study comparisons challenging.

MusD predicted both physical performance and self‐reported physical function independent of muscle quantity (i.e., ALM/height^2^). Grip strength was ~3% greater for every 5% greater forearm MusD, and every 10% greater leg MusD was associated with ~1% greater grip strength, fast gait speed and PROMIS‐PF score. These data further those of Erlandson et al. [14] and Chow et al. [27] who used first‐generation HR‐pQCT scanners to show that MusD was associated with physical performance in postmenopausal women and women following long bone fracture, respectively. However, these previous studies did not control for the impact of muscle quantity, and they acquired HR‐pQCT outcomes at the distal end of the tibia—a location that includes a large proportion of nonmuscular tendinous structures, including the Achilles tendon.

Most recently, Orwoll et al. [15] demonstrated that MusD acquired by a second‐generation HR‐pQCT scanner at the same leg location as in the current study was independently associated with physical performance—namely, grip strength, usual gait speed and the number of chair stands completed in 10 s. Our data support these observations, with leg MusD predicting grip strength (as well as fast gait speed and self‐reported physical function), but we did not quite reach significance for usual gait speed (*p* = 0.10) or the number of sit‐to‐stand manoeuvres completed in 30 s (*p* = 0.09). The prediction of different physical performance outcomes between studies may relate to idiosyncrasies in physical function test execution and study populations, with Orwoll et al. [15] studying older men all aged between 77 and 101 years.

In addition to predicting physical performance, Orwoll et al. [15] demonstrated that HR‐pQCT acquired MusD was associated with the risk of mobility limitation and mobility disability and the risk of mortality. These observations were independent of muscle mass (assessed by D_3_‐creatine dilution) and total body fat, confirming observations that MusD provides novel information regarding muscle and general health [5, 7, 32] To facilitate further utility of HR‐pQCT acquired MusD, we generated reference data and developed a calculator that provides standardised data in the form of age‐ and sex‐matched *z*‐scores (and their associated percentiles) and sex‐specific *t*‐scores (i.e., MusD standardised to young healthy adults).

The calculator provides clinicians and investigators a means of expressing data from an individual or population of interest relative to our reference cohort. Its use may negate the need to establish a control group in cross‐sectional studies and can indicate the magnitude of difference from expected values. To demonstrate the utility of the calculator, we entered data acquired from female collegiate‐level athletes (*n* = 50) and individuals with CKD undergoing dialysis (*n* = 50). As expected from studies demonstrating that exercise increases MusD [37], the athletes had above‐average leg MusD; however, the difference was not large (*z*‐score = 0.20; 95% CI, 0.01–0.39). In contrast, individuals with end‐stage CKD had substantially reduced MusD at both the forearm and leg (*z*‐scores <−1.5) supporting studies reporting on compromised muscle quality in this population [38].

Our study has several strengths but is not without limitations. Data were obtained cross‐sectionally. There is potential for secular differences between generations, and the reference curves may not represent an individual's trajectory over time. Data were obtained in a mostly (85%) White population from the Midwest of the United States using a single scanner. The potential for racial, ethnic, geographic and between‐machine differences requires consideration. The scan location was isolated to a small axial length (10.2 mm) of muscle located at 30% of bone length proximal from the distal end of the radius and tibia. These locations are readily accessible in most individuals with routine use of the second‐generation scanner but may not be representative of outcomes at other sites, with muscle fat infiltration potentially being inhomogeneous between muscles and along muscle length. The potential for differences in MusD between sites is evident in the current data, with forearm MusD explaining only 23.4% and 24.1% of the variance in leg MusD in females and males, respectively (Pearson’s correlation coefficients: *r* = 0.48–0.49, all *p* < 0.001). The outcomes are specific to the scanning, segmentation and analysis procedures used. Also, we did not consider the potential impact of beam hardening and artifactual decrease in MusD in larger limbs due to the preferential absorption of low‐energy photons [39, 40]. Finally, we had more limited inclusion of males and were not able to provide reference data for individuals < 18 years or > 80 years of age.

In summary, the current study provides novel observations and reference data for MusD acquired using HR‐pQCT. Forearm and leg MusD provide information on physical performance and function that is unique from the information provided by a standard indicator of muscle quantity (i.e., ALM/height^2^). Whether the unique information provided by MusD has a role in quantifying health and the consequences of disease and illness (including sarcopenia and cachexia) requires further exploration. Studies in this area may be facilitated by the reference data generated herein, which enable HR‐pQCT MusD in an individual or population of interest to be expressed relative to the reference cohort.

## Ethics Statement

All procedures were approved by the Institutional Review Board of Indiana University and were performed in accordance with the ethical standards laid down in the 1964 Declaration of Helsinki and its later amendments. All participants provided written informed consent prior to their inclusion to participate and for their data to be utilised.

## Conflicts of Interest

The authors declare no conflicts of interest.

## Supporting information


**Data S1** **.** Supplementary Information

**Data S2** **.** Supplementary Information

**Data S3** **.** Supplementary Information

**Data S4** **.** Supplementary Information
