# Supplementary material for: Reference Data and Predictors of HR‐pQCT‐Derived Muscle Density and Its Prediction of Physical Performance
Source: J Cachexia Sarcopenia Muscle. 2025 Jul 30;16(4):e70029. doi: 10.1002/jcsm.70029 (PMC12308218; doi:10.1002/jcsm.70029)
Supplement: Supplementary file 1 — Data S1 . Supplementary Information [file JCSM-16-e70029-s003.pdf]

Supplemental material 1. Predictors of forearm and leg MV, TV, and MV/TV[a]

HR-pQCT muscle outcome & candidate predictors	Forearm				Leg			
	Estimate	SE	Adj. P	R^2	Estimate	SE	Adj. P	R^2
MV (cm^3)				0.787				0.522
Age (yr)	**0.009**	**0.004**	**0.02**		0.014	0.007	0.06	
Sex (female vs. male)	**-3.300**	**0.218**	**<0.001**		0.843	0.442	0.06	
Race								
Asian vs White	-0.439	0.239	0.07		0.299	0.480	0.53	
Black vs White	-0.143	0.223	0.52		**-4.029**	**0.463**	**<0.001**	
Others vs White	-0.176	0.782	0.82		-1.760	1.465	0.23	
Ethnicity (Hispanic vs. non-Hispanic)	-0.456	0.289	0.12		**-1.507**	**0.576**	**<0.01**	
Height (cm)	**0.044**	**0.009**	**<0.001**		**0.089**	**0.018**	**<0.001**	
BMI (kg/m^2)	**0.090**	**0.028**	**0.001**		0.097	0.060	0.11	
Percent body fat (%)	**-0.131**	**0.016**	**<0.001**		**-0.073**	**0.034**	**0.03**	
ALM/height^2 (kg/m^2)	**1.408**	**0.093**	**<0.001**		**2.523**	**0.200**	**<0.001**	
TV (cm^3)				0.763				0.469
Age (yr)	0.006	0.005	0.23		-0.017	0.011	0.12	
Sex (female vs. male)	**-3.525**	**0.274**	**<0.001**		**3.188**	**0.660**	**<0.001**	
Race								
Asian vs White	-0.281	0.300	0.35		-0.476	0.716	0.51	
Black vs White	**-0.949**	**0.28**	**<0.001**		**-6.276**	**0.690**	**<0.001**	
Others vs White	-0.143	0.981	0.88		-1.972	2.186	0.37	
Ethnicity (Hispanic vs. non-Hispanic)	-0.606	0.363	0.10		**-2.180**	**0.859**	**0.01**	
Height (cm)	**0.078**	**0.011**	**<0.001**		**0.146**	**0.027**	**<0.001**	
BMI (kg/m^2)	**0.216**	**0.035**	**<0.001**		**0.346**	**0.090**	**<0.001**	
Percent body fat (%)	-0.024	0.021	0.24		0.097	0.051	0.06	
ALM/height^2 (kg/m^2)	**1.691**	**0.117**	**<0.001**		**3.005**	**0.298**	**<0.001**	
MV/TV (%)				0.604				0.416
Age (yr)	**0.020**	**0.008**	**0.01**		**0.070**	**0.010**	**<0.001**	
Sex (female vs. male)	**-2.136**	**0.454**	**<0.001**		**-2.789**	**0.595**	**<0.001**	
Race								
Asian vs White	**-1.020**	**0.497**	**0.04**		**1.484**	**0.645**	**0.02**	
Black vs White	**2.549**	**0.465**	**<0.001**		0.687	0.622	0.27	
Others vs White	-0.156	1.629	0.92		-1.830	1.970	0.35	
Ethnicity (Hispanic vs. non-Hispanic)	0.469	0.602	0.44		0.375	0.774	0.63	
Height (cm)	**-0.057**	**0.018**	**<0.01**		-0.024	0.025	0.33	
BMI (kg/m^2)	-0.087	0.057	0.13		-0.133	0.081	0.10	
Whole-body percent fat (%)	**-0.608**	**0.034**	**<0.001**		**-0.433**	**0.046**	**<0.001**	
ALM/height^2 (kg/m^2)	-0.140	0.190	0.47		0.465	0.268	0.08	

ALM = appendicular lean mass; BMI = body mass index; FatD = fat density; MusD = muscle density; MV = muscle volume; MV/TV = muscle volume/total volume; SE = standard error; TV = total volume

[a]Determined using multivariable linear regression (enter method)
