# Supplementary material for: Reference Data and Predictors of HR‐pQCT‐Derived Muscle Density and Its Prediction of Physical Performance
Source: J Cachexia Sarcopenia Muscle. 2025 Jul 30;16(4):e70029. doi: 10.1002/jcsm.70029 (PMC12308218; doi:10.1002/jcsm.70029)

Supplementary material 2

Raw data and fitted centile curves for forearm (A,B) and leg (C,D) muscle volume (MV) in females (A,C) and males (B,D).

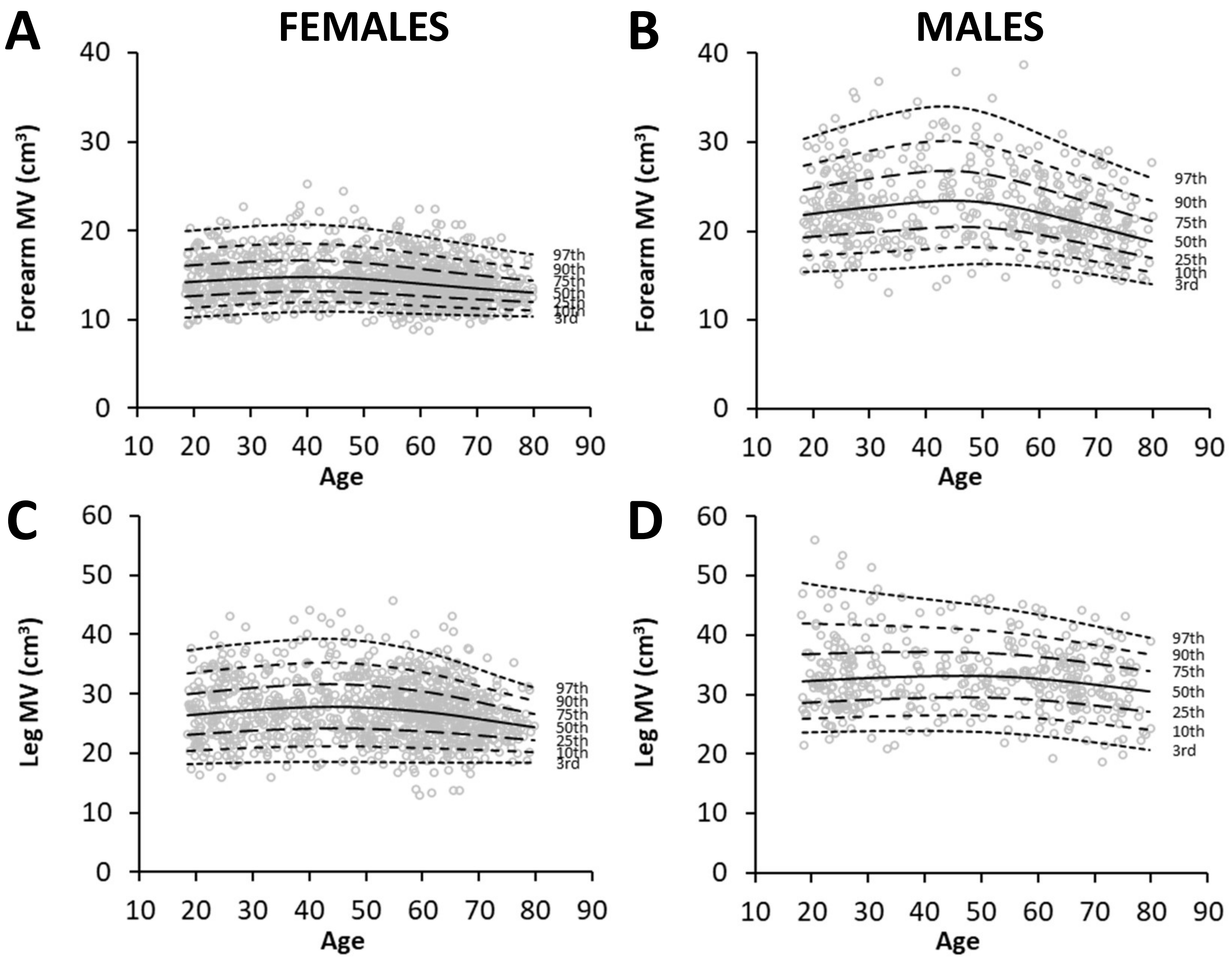

Raw data and fitted centile curves for forearm (A,B) and leg (C,D) tissue volume (MV) in females (A,C) and males (B,D).

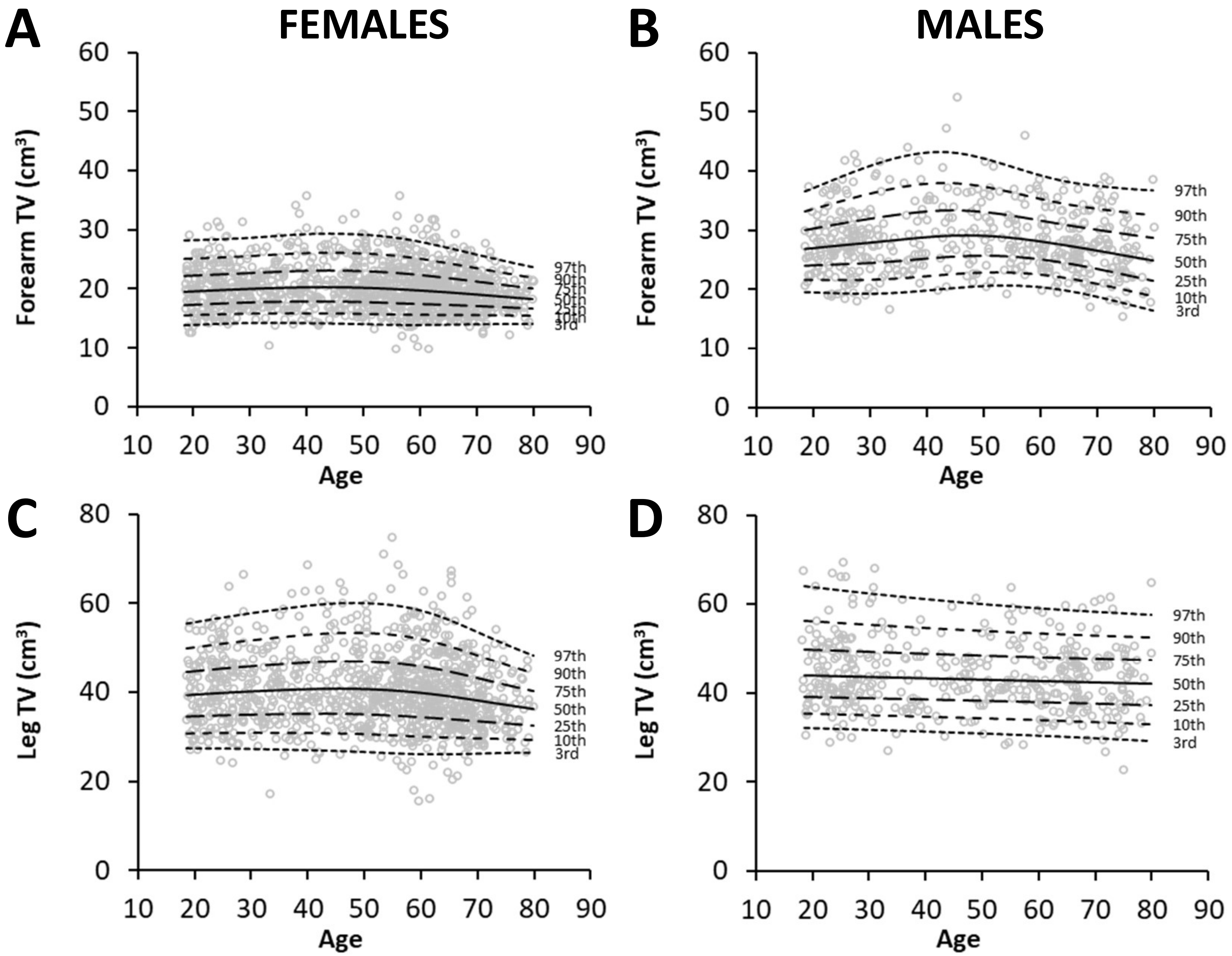

Raw data and fitted centile curves for forearm (A,B) and leg (C,D) muscle volume fraction (MV/TV) in females (A,C) and males (B,D).

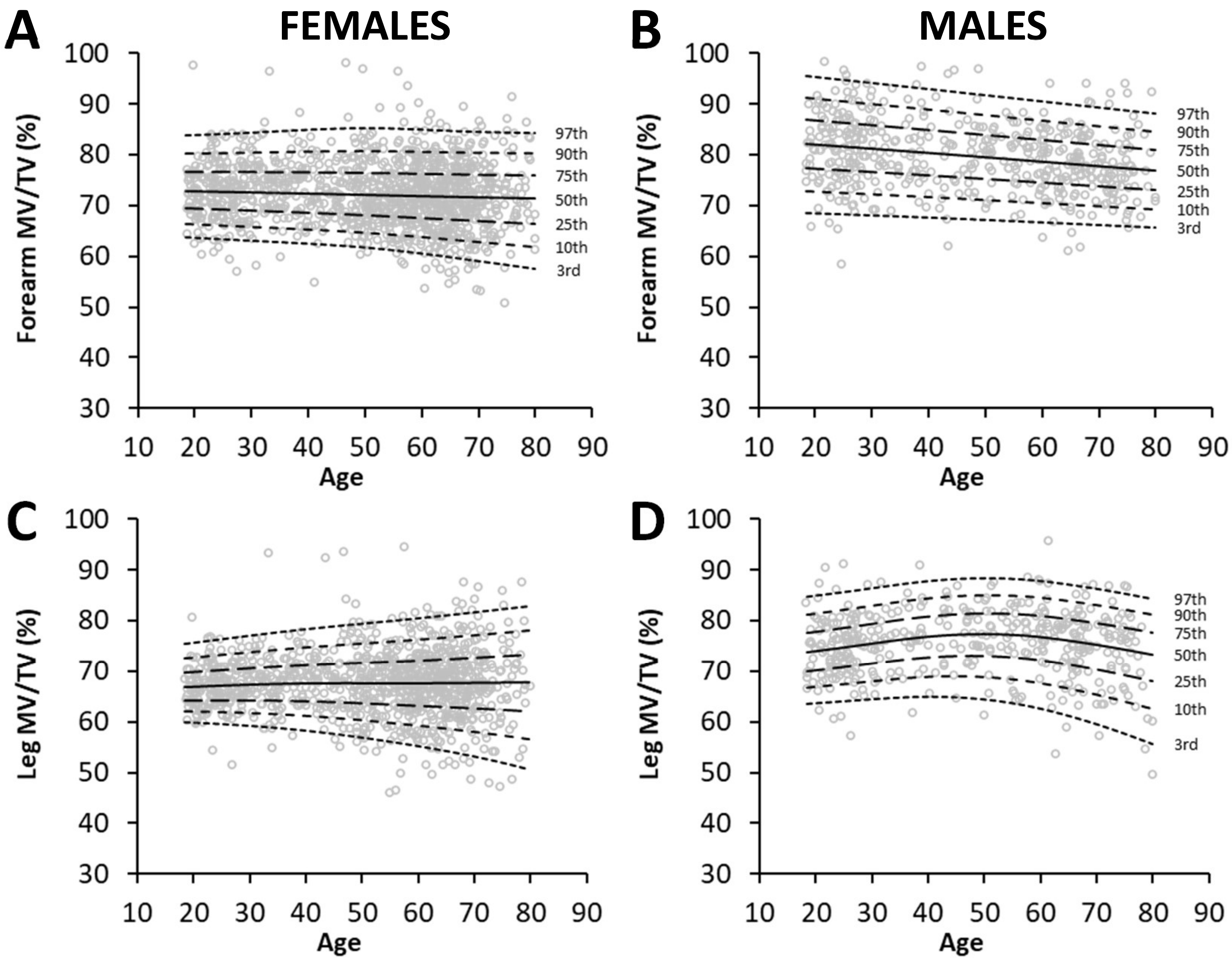

Supplement: Supplementary file 2 — Data S2 . Supplementary Information [file JCSM-16-e70029-s001.pdf]
