# Supplementary material for: Reference Data and Predictors of HR‐pQCT‐Derived Muscle Density and Its Prediction of Physical Performance
Source: J Cachexia Sarcopenia Muscle. 2025 Jul 30;16(4):e70029. doi: 10.1002/jcsm.70029 (PMC12308218; doi:10.1002/jcsm.70029)
Supplement: Supplementary file 4 — Data S4 . Supplementary Information [file JCSM-16-e70029-s004.pdf]

Suppplemental material 4. Prediction of physical performance and function by MV/TV (%)[a]

Physical function outcome	Base model[b]	Base model + forearm MV/TV				Base model + leg MV/TV			
	R^2	R^2	Estimate[c]	SE[c]	Adj. P[c]	R^2	Estimate[c]	SE[c]	Adj. P[c]
Usual gait speed (m/s)	0.128	0.128	0.001	0.001	0.83	**0.133**	**0.003**	**0.001**	**<0.01**
Fast gait speed (m/s)	0.228	0.230	-0.002	0.002	0.21	**0.234**	**0.004**	**0.001**	**<0.01**
Grip strength (kg)	0.648	**0.651**	**0.131**	**0.041**	**<0.01**	**0.651**	**0.112**	**0.034**	**<0.001**
30-s sit-to-stand test (n)	0.259	0.261	-0.013	0.022	0.54	0.261	0.005	0.018	0.80
PROMIS-PF (T-score)	0.374	0.375	-0.007	0.039	0.85	0.375	0.002	0.032	0.95

MV/TV = muscle volume/tissue volume; PROMIS-PF = physical function domain of the National Institutes of Health Patient Reported Outcomes Measurement Information System; SE = standard error

[a]Determined using multivariable linear regression

[b]Base model predictors included were age, sex, race, ethnicity, height, BMI, whole-body percent fat, and appendicular lean mass/height^2

[c]Estimate, SE and adjusted (adj.) P-value are for HR-pQCT soft tissue composition after controlling for the influence of base model predictors
